# Sarcoidosis with musculoskeletal manifestations: systematic review of non-pharmacological and pharmacological treatments

**DOI:** 10.1007/s00296-022-05171-8

**Published:** 2022-08-09

**Authors:** Geir Smedslund, Annie Martina Kotar, Till Uhlig

**Affiliations:** 1 Division of Rheumatology and Research, Diakonhjemmet Hospital, Norwegian National Advisory Unit On Rehabilitation in Rheumatology, Oslo, Norway; 2 University of Oslo, Oslo, Norway

**Keywords:** Sarcoidosis, Musculo-skeletal, Systematic review, Lӧfgren’s syndrome

## Abstract

We aimed to summarise effects and use of non-pharmacological and pharmacological treatments for sarcoidosis with musculoskeletal manifestations. We systematically searched the Cochrane Library, Ovid MEDLINE, Embase, CINAHL, AMED, Scopus, clinical.trials.gov, PROSPERO and PEDro for systematic reviews from 2014 to 2022 and for primary studies from date of inception to March 29, 2022, and studies with patients diagnosed with sarcoidosis with musculoskeletal manifestations. Inclusion criteria required that studies reported effects of non-pharmacological and/or pharmacological treatments or number of patients receiving these treatments. Results were reported narratively and in forest plots. Eleven studies were included. No systematic reviews fulfilled our inclusion criteria. None of the included studies had a control group. We found that between 23 and 100% received corticosteroids, 0–100% received NSAIDs, 5–100% received hydroxychloroquine, 12–100% received methotrexate, 0–100% received TNF inhibitors, and 3–4% received azathioprine. Only ten patients in one study had used non-pharmacological treatments, including occupational therapy, chiropractic and acupuncture. There are no controlled studies on treatment effects for patients with sarcoidosis with musculoskeletal manifestations. We found 11 studies reporting use of pharmacological treatments and only one study reporting use of non-pharmacological treatments. Our study identified major research gaps for pharmacological and non-pharmacological treatment in musculoskeletal sarcoidosis and warrant randomised clinical trials for both.

## Introduction

Sarcoidosis is a rare disease of unknown aetiology which can affect several organs [1]. Prevalence and presenting symptoms of sarcoidosis vary significantly by sex, racial group, and country, and in the United States, an age-adjusted annual incidence of 35.5 per 100,000 for blacks was observed as compared with 10.9 per 100,000 for whites [2].

In most cases, the respiratory system is involved [3], other affected organ systems are skin, eyes, and systemic symptoms, such as fever, night sweats, fatigue, and malaise. Within the musculoskeletal system, ankles, knees and wrists are the most commonly involved joints, with oligo-articular involvement more prominent in acute arthritis [4].

Löfgren’s syndrome is a typical musculoskeletal manifestation with acute onset of Erythema nodosum with periarticular ankle inflammation together with hilar lymphadenopathy and is associated with a benign disease course [5]. Myopathy can result in pain and muscle weakness, whilst chronic arthropathy and osseous manifestations are rather uncommon [1].

The clinical presentation of sarcoidosis can be quite variable, as can the prognosis, ranging from mild and self-limited to severe disease that leads to significant organ impairment and increased mortality [6]. Chronic progressive disease may require long-term treatment with corticosteroids, cytotoxics and other agents which have a potential for considerable adverse events [3].

Glucocorticosteroids are considered first line treatment in sarcoidosis [1, 7]. Disease-modifying antirheumatic drugs, such as methotrexate, azathiorine, hydroxychloroquine, or a tumour necrosis factor inhibitor (TNFi or TNF inhibitors), have been used successfully and in combination with corticosteroids [8]. In a recent review, TNFi was given in 12% of patients with sarcoidosis in rheumatology practices in the United States [9].

The disease requires cooperation between specialists, but the role of non-pharmacological management has not been a topic of interest in the literature for patients with musculoskeletal manifestations as compared to rehabilitation supported in pulmonary sarcoidosis [10].

The aim of this study was to systematically review the literature on sarcoidosis with musculoskeletal manifestations for evidence of non-pharmacological and pharmacological treatment effects. Results will inform clinicians on evidence-based pharmacological and non-pharmacological treatment decisions in patients with musculoskeletal manifestations of sarcoidosis. The review will also identify research gaps. If no effect studies were found, we included studies with reported use of specified treatments. This review was not registered in a prospective register of systematic reviews.

## Materials and methods

### Inclusion criteria

We included patients with sarcoidosis with joint and bone manifestations, including Löfgren’s syndrome. We searched primarily for systematic reviews. If no systematic reviews were found, we searched for primary studies with or without a control group. Included designs were: Randomised controlled trials (including cluster-randomised, quasi-randomised, stepped wedge), regression discontinuity design, controlled clinical trials, longitudinal studies, case series, and cohort studies. Excluded designs were qualitative studies, case–control studies, cross-sectional studies and case studies, and publications in abstract format. Our PICO (patients-interventions-comparisons-outcomes) for the effect question was:

P: Patients with sarcoidosis with joint and bone manifestations, including Löfgren’s syndrome.

I: Non-pharmacological and pharmacological interventions.

C: Standard care, placebo, other active non-pharmacological or pharmacological intervention.

O: All outcomes.

If we did not find effect studies, we had the same ‘P’ and ‘I’ but aimed to describe the use of non-pharmacological and pharmacological interventions.

### Literature search

First, we performed a search for systematic reviews. The search for systematic reviews (in March 2022) was conducted in Cochrane Library, MEDLINE, Embase, Scopus and CINAHL, using a basic search strategy including keywords and MeSH-terms for patient group only. Thereafter, we searched for primary studies in Ovid MEDLINE(R) and Epub Ahead of Print, In-Process, In-Data-Review & Other Non-Indexed Citations and Daily < 1946 to March 28, 2022 > , Embase < 1974 to 2022 March 28 > , CINAHL and Scopus, using a more extensive search strategy. The full search strategies for MEDLINE and Embase are presented in Appendix 1.

We did not apply any language restrictions in the literature search. We included only studies in English. A librarian (AMK) performed the literature searches.

Two authors (GS and TU) independently screened titles/ abstracts and full texts according to the inclusion criteria. One author (GS) extracted data, and the other author (TU) checked the data extractions. For appraisal of methodological quality, we used the Joanna Briggs Case Series Critical Appraisal Tool [11]. The tool has 10 domains on which to assess risk of bias for each study (Appendix 3). The tool does not have a sum score, so it is not feasible to assess the within-study risk of bias. For a similar reason, we did not assess the level of risk of bias per medication/ medication group. One author (GS) performed the appraisal, and the second author (TU) checked the appraisal. We planned to perform meta-analyses of effect studies. If no such studies were found, we considered to perform meta-analyses of the proportion of patients that used different types of non-pharmacological or pharmacological treatments in the studies. A prerequisite for performing meta-analyses is that studies are sufficiently homogeneous with respect to patients, medications, outcomes, and study designs. Studies with low methodological quality were not judged to be eligible for meta-analysis. We also supply a narrative description of the results of each included study.

### Statistical analysis

We constructed forest plots with the R package “meta” and the command “metaprop”.[12]. We present plots on the proportion of patients in each study who used the different kinds of medications. We present one plot for each specified medication (corticosteroids, non-steroidal anti-inflammatory drugs (NSAIDs), hydroxychloroquine, methotrexate, TNF inhibitors, and azathioprine).

## Results

We identified five reviews [1, 13–16], none of which were fulfilling our inclusion criteria. The review by Shariatmaghani et al. [1] was not a systematic review. Adler [13] studied effects of TNF inhibitors on sarcoidosis, but there were no patients with musculoskeletal manifestations. Another (non-systematic) review by Baughman [14] proposed a treatment scheme but did not report treatment effects. A fourth review by Bechman [15] was not a systematic review and did not report treatment effects. Finally, the article by Drent [16] was not a systematic review.

**Fig. 1:**
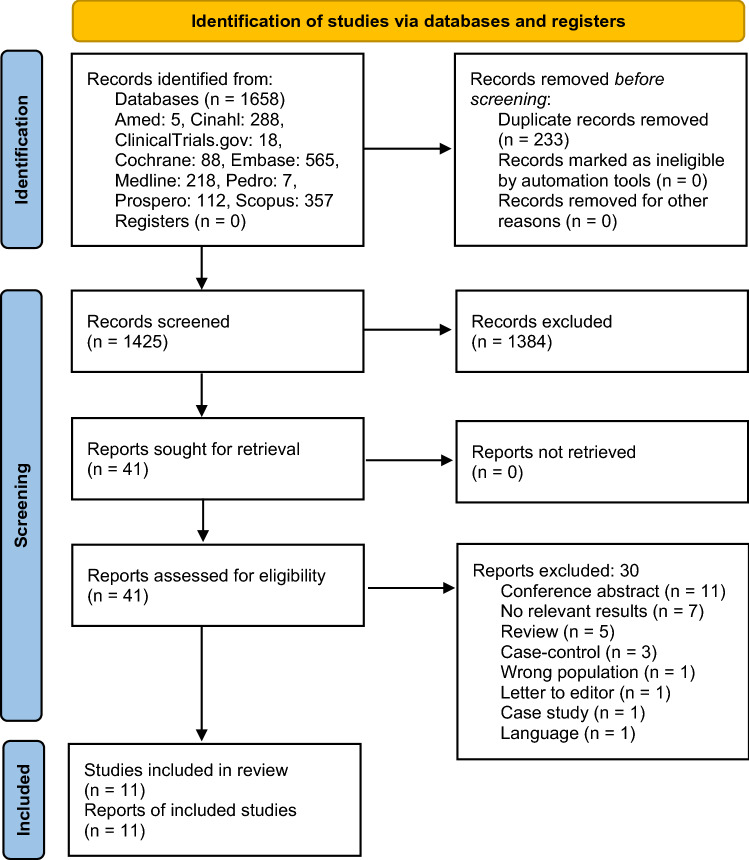
PRISMA_2020_flow_diagram. *From:* Page MJ, McKenzie JE, Bossuyt PM, Boutron I, Hoffmann TC, Mulrow CD, et al. The PRISMA 2020 statement: an updated guideline for reporting systematic reviews. BMJ 2021;372:n71. https://doi.org/10.1136/bmj.n71. For more information, visit:http://www.prisma-statement.org/

The combined search for systematic reviews and primary studies found 1425 unique records after removal of duplicates. The flow chart (Fig. 1) shows the inclusion process. We obtained 41 articles in full text and included 11 articles [4, 17–26], which are listed in Table 1. One article [53] was in Spanish and was therefore excluded. The 30 excluded articles [5, 9, 27–54] are listed in Appendix 2.

**Table 1:** Included studies (*n* = 11)

Author/year (country)	Design	Patients (n, gender, mean age)	Disease manifestation	Focus of attention	Outcomes
Arthritis in Sarcoidosis Group 2018 (India)	Retrospective case records	*N* = 117, 49% males, age = 40	Sarcoid arthritis (some with Löfgren’s syndrome)	Proportion with Löfgren’s syndrome, pattern of joint involvement	Peripheral lymphadenopathy, uveitis, remission, mortality
Banse 2013 (France)	Retrospective, one group, before-after study	*N* = 10, 40% males, age = 43	Joint manifestations of sarcoidosis	Effect of TNF inhibitors	No of painful and swollen joints, DAS28 ESR, CRP, VAS, EULAR score
Cacciatore 2020 (France)	Retrospective multi-centre study	*N* = 39, 36% males, age = 41	Sarcoid arthropathy	Differential diagnoses and prevalence of symptoms	NSAIDs, steroids, methotrexate, hydroxychloroquine or TNF inhibitors
Fayad 2006 (France)	Retrospective medical chart review	*N* = 5, 40% men, age = 49	Sarcoidosis with muscle involvement	Description of features and outcomes	Medication use, nodular vs. myositic type, cardiac and renal relapse, steroid dosage, muscle relapse
Garg 2009 (India)	Case series	*N* = 18, 56% males, age = 33	Acute inflammatory ankle arthritis	Classification Löfgren/Ponchet	Differential diagnosis (Löfgren’s syndrome/Ponchet’s disease
Glennås 1995 (Norway)	Prospective cohort	*N* = 17, 71% males, age = 30	Acute sarcoid arthritis	Occurrence, seasonal onset, clinical features, outcome	Remission, duration of arthritis, erythema nodosum, lung and hilar manifestations
Loupasakis 2015 (USA)	Case series	*N* = 11, 100% males, age = 43	Refractory sarcoid arthritis	Describe cases of sarcoid arthritis in firefighters	Prognosis
Mana 2003 (Spain)	Case series	*N* = 17, 94% males, age = 40	Recurrent sarcoidosis (Löfgren)	Investigate clinical characteristics	Recurrence
Miller 2019 (USA)	Retrospective cohort study	*N* = 24, 46% males, age = 50	Osseous sarcoidosis	Musculo-skeletal and pulmonary outcomes	Use of glucocorticoids and DMARDs
Perruquet 1984 (USA)	Retrospective review	*N* = 32, 22% males, age = 34	Sarcoid arthritis	Prognosis	Incidence of joint involvement
Petursdottir 2007 (Iceland)	Cohort from national registry	*N* = 39, 41% males, age = 54	Sarcoidosis with skeletal symptoms	Prevalence and prognosis	Recovery

Characteristics of included studies Table 1 shows characteristics of the included studies. They were published between 1984 and 2020. Three studies were from France, three were from USA, two from India, and the remainder from Norway, Spain, and Iceland. No studies had a control group, and none reported effects of treatment. The total number of patients was 329. The number of patients in individual studies varied between 5 and 117. The mean per cent males were 54.1 (the mean of the means in each study), and ranged from 22 to 100. Mean age in the studies was 41.5 (standard deviation: 7.4) years. The aims of the studies were mostly to describe occurrence of different diagnoses and symptoms and/or describe treatments given to patients. One study (Garg [20]) tried to improve on the classification to Löfgren’s syndrome or Ponchet’s disease. Banse [17] studied the effects of TNF inhibitors. Glennås [21] explored seasonal variations of disease onset.

*Methodological quality appraisal.* Only two of the studies [21, 23] were assessed as fulfilling all the quality items in the quality appraisal, whilst six studies had serious shortcomings. The most common shortcomings were lack of consecutive inclusion, unclear reporting of outcomes or follow-up, and unclear reporting of the presenting site(s)/clinic(s) demographic information. Appendix 3 is a description of how we assessed the included studies on methodological quality.

### Description of the studies

*Arthritis in Sarcoidosis Group 2018* recruited patients from 11 centres in India. The medical records from these centres were reviewed to locate patients with sarcoidosis who had arthritis from 2005 onward. Patients were categorised into acute sarcoid arthritis (≤ 6 months) or chronic sarcoid arthritis (> 6 months). The authors compared acute and chronic sarcoid arthritis groups for differences in demographic profile. Of 117 patients, 88 had acute and 29 had chronic sarcoid arthritis. Forty-five patients in the acute group had Löfgren’s syndrome, and one patient in the chronic group had Heerfordt’s syndrome. *Treatments given:* All patients had received non-steroidal anti‐inflammatory drugs. For extra‐articular manifestations, 35 received corticosteroids, 17 received weekly methotrexate, 12 were on hydroxychloroquine and three on azathioprine. *Prognosis***:** About four‐fifths of the 49 patients with acute sarcoid arthritis followed up for a median of about 2 years had attained complete remission with non‐steroidal anti‐inflammatory drugs, with corticosteroids and other DMARDs used for extra‐articular features. Acute sarcoid arthritis is mostly self‐limiting. *Conclusions***:** The authors concluded that ankles, knees and wrists are the most commonly involved joints, with oligo-articular involvement more prominent in acute arthritis as opposed to similar oligo‐ and poly-articular involvement in chronic arthritis. Both acute and chronic articular sarcoidosis have greater propensity for hilar involvement, ILD and erythema nodosum. However, uveitis and peripheral adenopathy are more common in chronic sarcoid arthritis.

*Banse 2013* evaluated the efficacy and safety of three TNF inhibitors to treat joint manifestations of sarcoidosis. Included patients were refractory to conventional therapy (NSAIDs, corticosteroids, and/or disease-modifying antirheumatic drugs (DMARDs). Amongst the ten patients, five had arthralgias without swelling and five had arthritis, including two mono-, one oligo-, and two polyarthritis. Before initiation of TNF inhibitors, according to disease activity (DAS28-CRP) scores, six patients had low articular disease activity (DAS28 < 3.2), two had moderate (DAS28 3.2–5.1), and two had high disease activity (DAS28 > 5.1). *Treatments given:* All patients received TNF inhibitors, and the results were reported after 3, 6, and 12 months. The total duration of TNF inhibitor exposure was 17.6 patient-years, which was started a median of 3 (0.33–17) years after sarcoidosis diagnosis. Infliximab, adalimumab and etanercept was first- and second-/third-line choices, respectively. *Conclusions***:** The authors concluded that no significant impact of a TNF inhibitor on articular manifestations (numbers of painful and swollen joints, DAS28 with ESR or CRP, global VAS score), extra-articular involvement (pulmonary, ocular, cardiac, muscular), or biological indicators of inflammatory syndrome were observed. However, TNF inhibitor use obtained significant, albeit moderate, corticosteroid sparing effects. Moreover, their safety seems good, with no severe adverse events occurring under treatment.

*Cacciatore 2020* included 39 patients with sarcoid arthropathy in a retrospective study. They contrasted 19 patients with acute disease (Löfgren’s syndrome) with 20 chronic patients. *Treatments given***:** Amongst 20 patients with chronic sarcoidosis, treatment was used in 17 cases, and they used either NSAIDs alone (*n* = 5), steroids alone (*n* = 5), hydroxychloroquine (*n* = 2), methotrexate (*n* = 3), or TNF inhibitors (*n* = 2). *Conclusions:* Sarcoid arthropathies have different clinical phenotypes in acute and chronic forms and various treatment regimens, such as hydroxychloroquine and methotrexate, could be used in chronic forms.

*Fayad 2006* performed a retrospective study from hospital charts over the period 1985–2001 in two academic French rheumatology centres. Five patients with muscle sarcoidosis were included. *Treatments given:* All patients were given prednisolone and two patients used hydroxychloroquine. *Conclusion:* Symptomatic muscle involvement may be an initial feature of chronic, and usually the systemic form of, sarcoidosis. It responds to corticosteroid therapy, but relapse seems to be frequent.

*Garg 2009* examined patients with acute inflammatory ankle arthritis to establish whether they had Löfgren’s syndrome (acute presentation of sarcoidosis) or Ponchet’s disease (reactive arthritis due to tuberculosis infection). They also presented an algorithm for differential diagnosis of such patients. Of 18 patients, 10 were classified with Löfgren’s syndrome, and all of them had a negative Mantoux test (computerised tomography). The remaining patients were classified with Ponchet’s disease, all of them had a positive Mantoux test. *Treatments received:* The patients also received drug treatment (glucocorticoids and glucocorticoid-sparing drug methotrexate and hydroxychloroquine in patients with Löfgren’s syndrome and anti-tuberculosis drugs in Ponchet’s disease. The numbers treated with each drug were not reported.). *Prognosis:* All patients with Löfgren’s syndrome responded rapidly to the drugs and became symptom-free over a period of 8–12 weeks. The authors reported that all the patients with Ponchet’s disease responded satisfactorily with complete clinical as well as radiological response. *Conclusions:* The algorithm was successful at distinguishing between Löfgren’s syndrome and Ponchet’s disease.

*Glennås 1995* followed 186 patients presenting with acute arthritis and with suspicion for reactive arthritis for 2 years and sought to classify according to diagnosis. The number of new cases of sarcoid arthritis (SA) per year in Oslo was 2.9/100 000 persons between 18 and 60 years of age. The authors found a clustered onset of SA in the spring (February-June, *p* = 0.01). All 17 cases of SA had complete remission of arthritis at the 104-week follow-up. *Treatments received:* Eight of eleven patients received corticosteroids, and 13 of 17 received NSAIDs. *Conclusions:* The authors concluded that the outcome of acute sarcoid arthritis appeared favourable.

*Loupasakis 2015* described a case series with sarcoid arthritis in eleven firefighters in New York who worked at the World Trade Center (WTC) site on September 11, 2001. Nine of 60 firefighters who developed sarcoidosis after this date presented with poly-articular arthritis. Two others diagnosed pre-9/11/2001 developed sarcoid arthritis post-WTC-exposure. All eleven were never cigarette smokers and all performed rescue/recovery at the WTC-site within three days of the attacks. All had biopsy-proven pulmonary sarcoidosis. *Treatments received:* All required additional disease-modifying anti-rheumatic drugs (DMARDs) for adequate control (stepwise progression from hydroxychloroquine to methotrexate to TNF inhibitors) of their joint manifestations. *Conclusions:* The authors concluded that chronic inflammatory polyarthritis appears to be an important manifestation of sarcoidosis in firefighters with sarcoidosis and WTC-exposure. Their arthritis is chronic, and unlike arthritis in non-WTC-exposed sarcoid patients, inadequately responsive to conventional oral DMARDs, often requiring TNF inhibitors.

*Mana 2003* studied recurrence of sarcoidosis following complete remission in 17 patients from Barcelona, Spain. Sixteen of the patients were women, and they experienced a total of 24 recurrences. The mean follow-up time was 143 ± 80 months. Löfgren’s syndrome was present in 16 patients at onset and in all patients at recurrence. *Treatments given:* Five patients received corticosteroids. *Conclusions***:** The authors concluded that acute sarcoidosis, and particularly Löfgren’s syndrome, may recur many years after complete remission, and, in general, still has a good outcome.

*Miller 2019* retrospectively followed the musculoskeletal and pulmonary outcomes of 24 patients with osseous sarcoidosis. They collected 1-year follow-up and last follow-up outcomes after diagnosis. The authors constructed a composite outcome consisting of (1) osseous sarcoidosis symptoms, (2) musculoskeletal imaging of affected bone, (3) chest imaging, and (4) pulmonary function testing. *Treatments given:* Three patients were already being treated with DMARDs or glucocorticoids for other sarcoid manifestations at the time of the osseous diagnosis. Miller et al. noted that current DMARD or glucocorticoid use at baseline was associated with a lower proportion of patients with positive worsening composite outcome (22% vs. 60%, *p* = 0.10). *Conclusions***:** Most patients had a favourable outcome according to symptoms, musculoskeletal/chest imaging, and PFTs, even though only a minority were treated with glucocorticoids or DMARDs.

*Perruquet 1984* conducted a retrospective review of all records of patients with a diagnosis of sarcoidosis who were seen at one centre between 1972 and 1982. Thirty-two (21%) of 150 patients with sarcoidosis had articular symptoms. *Treatments given***:** Patients were given salicylates, NSAIDs, prednisone, and colchicine. *Conclusion***:** Joint symptoms were generally transient. Acute sarcoid arthritis has a favourable prognosis.

*Petursdottir 2007* used data from the Icelandic Sarcoidosis Study, which contains all tissue-verified cases of sarcoidosis in Iceland since 1981. Their aim was to elucidate the prevalence, clinical manifestations and long-term prognosis of sarcoid arthritis in a nationwide cohort. Forty-seven of 234 patients reported skeletal symptoms. *Treatments given***:** The authors reported that patients were taking NSAIDs, DMARDs and glucocorticosteroids. Of 39 participants, 10 had used physiotherapy or occupational therapy. Three patients had used chiropractic treatment, and one had used acupuncture. *Conclusion***:** Around a fifth of all sarcoidosis patients develop joint symptoms, most frequently in the ankle. The prognosis is mostly favourable, but a subgroup of female patients may develop chronic polyarthritis.

Because the studies were heterogeneous and the methodological quality was so low, we decided to not perform meta-analyses, but present the results as forest plots.

Treatments received in each study.

### Pharmacological treatments

**Fig. 2:**
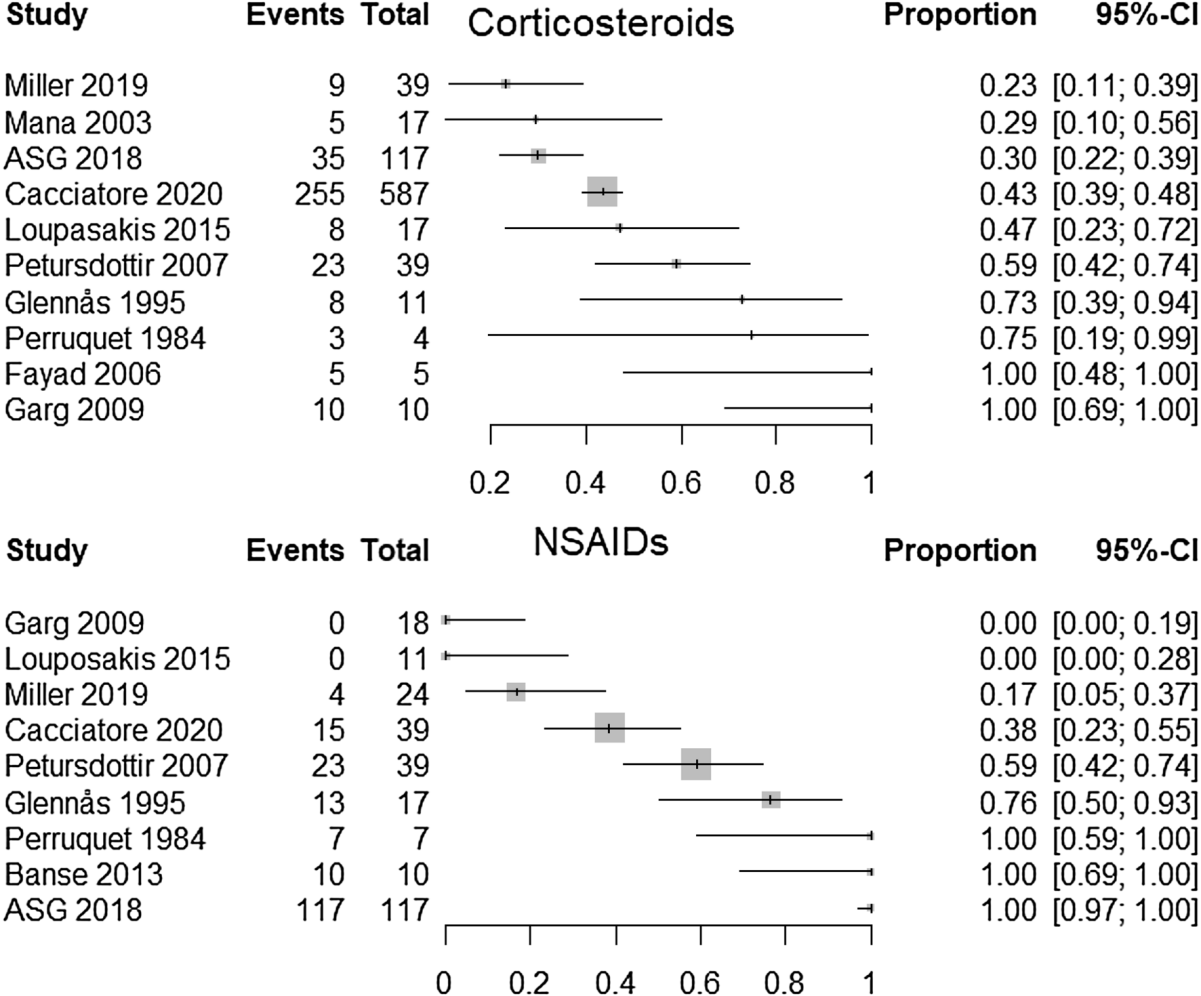
Forest plots of the proportion that received corticosteroids (upper panel) and NSAIDs (lower panel)

Figure 2 is a forest plot of the proportion of patients receiving corticosteroids and NSAIDs in each study. The upper panel shows that between 23 and 100 per cent received corticosteroids in the studies. The lower panel shows that between 0 and 100% received NSAIDs. Hence, the figures show substantial variation amongst studies.

**Fig. 3:**
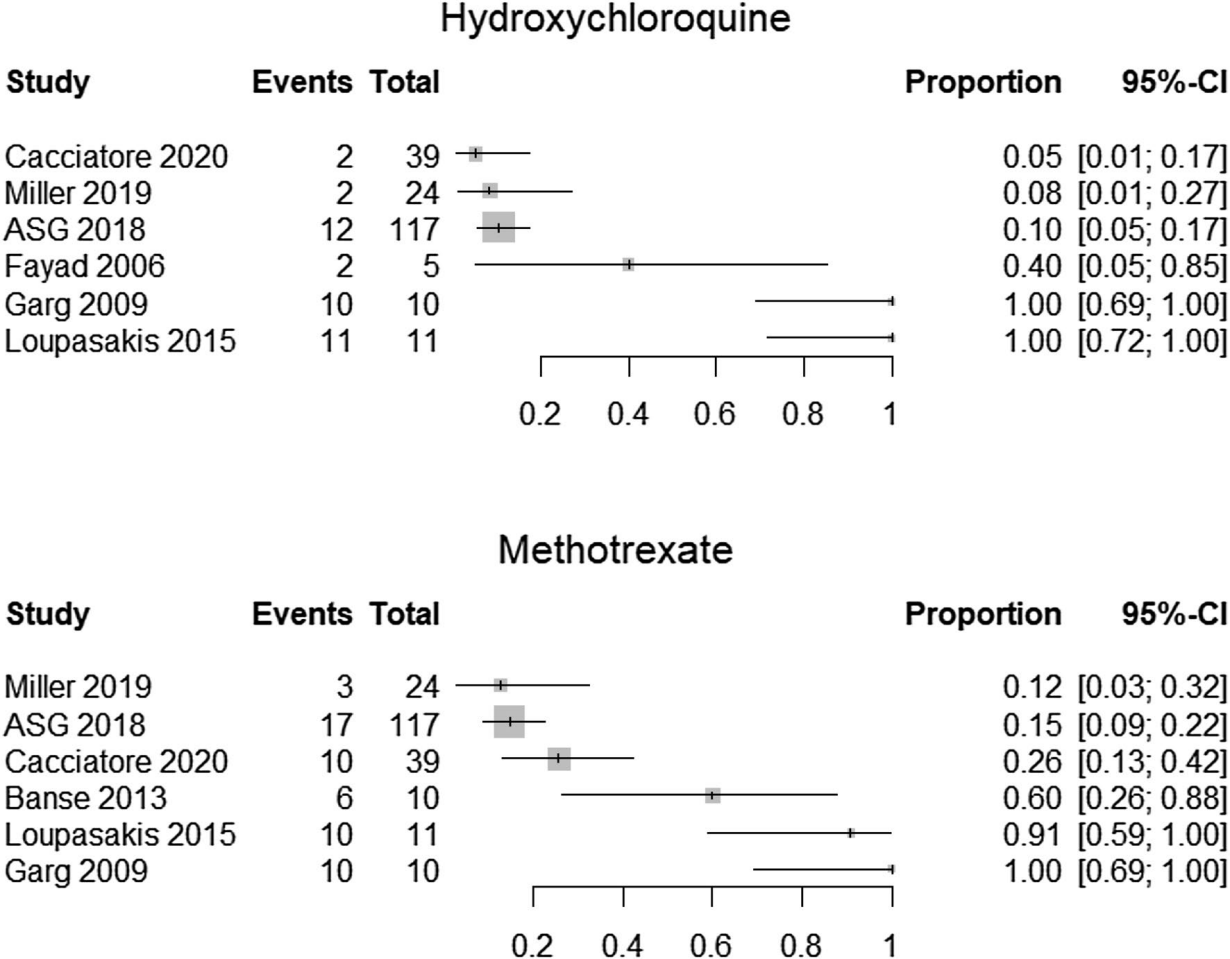
Forest plots of the proportion that received DMARDS; hydroxychloroquine (upper panel and methotrexate (lower panel)

Figure 3 shows the use of two DMARDs (hydroxychloroquine and methotrexate) in the same way. It is estimated that between 5 and 100% received hydroxychloroquine in the studies and that between 12 and 100% received methotrexate.

**Fig. 4:**
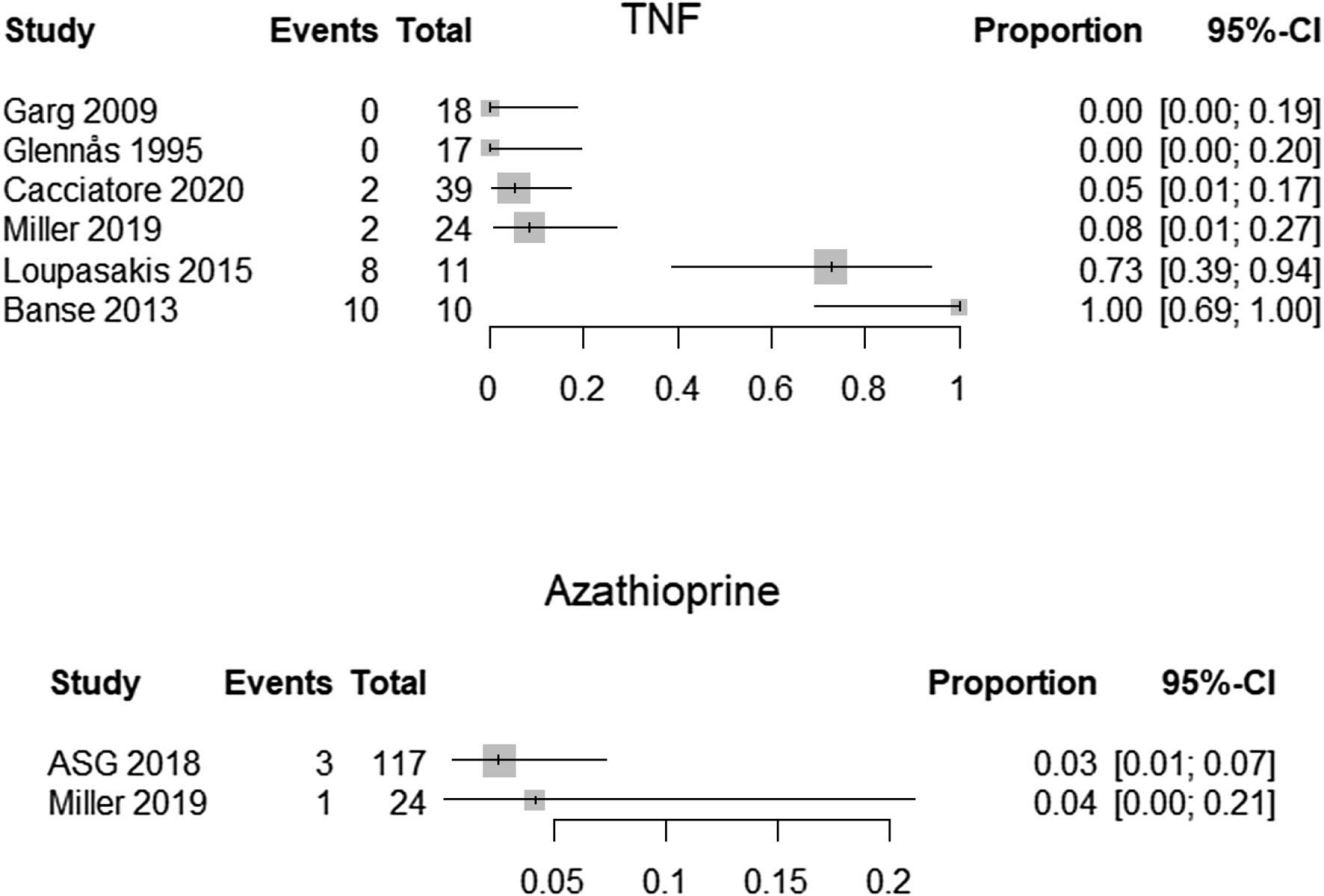
Forest plots of the proportion that received TNF inhibitors (upper panel) and Azathioprine (lower panel)

Figure 4 shows the use of TNF inhibitors (upper panel) and of azathioprine (lower panel). It is estimated that between 0 and 100% received TNF inhibitors, and that between 3 and 4% received azathioprine. There were, however, only two studies with a total of four patients that used azathioprine.

### Other pharmacological treatments

Petursdottir 2007 [26] reported that six participants had used DMARDs following diagnosis, whilst five had used these medications at the time of follow-up. The specific DMARDs used were not reported. Eleven participants in the study by Perruquet 1984 [25] had used salicylates, and two had used colchicine.

### Non-pharmacological treatments

Only the study by Petursdottir [26] reported use of various non-pharmacological treatments in altogether 10 patients.

## Discussion

We did not find any systematic review about non-pharmacological and pharmacological treatments in sarcoidosis with musculoskeletal manifestations. We, therefore, searched for primary studies, without finding a single study which had the aim to estimate treatment effects. Banse [17], Garg [20], and Miller [24] reported before-and-after data for patients using medications, but none of these studies were controlled. Hence, we cannot conclude about treatment effects for patients with musculoskeletal sarcoidosis. This is an important knowledge gap.

All of the ten included studies reported the number of patients using one medication or more. But because of large heterogeneity and poor methodological quality, we did not conduct meta-analyses for proportions of medications used. The forest plots showed large differences between studies in proportion of medication use and should be interpreted with caution. However, the plots give a visual representation of the pharmacotherapies provided in the included studies.

Thus, when clinicians decide on drugs for sarcoidosis, they have no specific evidence to guide them for what works and does not for this disease. Treatment alternatives may, thus, often be chosen with drugs that are known for their anti-inflammatory effects in other arthritic diseases, e.g. rheumatoid arthritis. Drugs against arthritis will therefore be used in this rare disease which may present with Löfgren’s syndrome as one form of reactive arthritis. It is surprising that no RCT for musculoskeletal manifestations of sarcoidosis has been performed. A reason for this could be that acute manifestations of musculoskeletal sarcoidosis are often successfully treated with medium doses of corticosteroids. If chronic manifestations develop, they may affect the musculoskeletal system in various areas and treatment will then be individualised. Another reason may be that frequent lung manifestations in sarcoidosis leave less focus on the chronic musculoskeletal disease burden.

In this systematic review, we included 10 studies and report proportions on the use of the following drug groups in the included studies: corticosteroids, NSAIDs, DMARDs (methotrexate and hydroxychloroquine), TNF inhibitors, and the immunosuppressive agent azathioprine. Because many patients were also participants in research studies with specific medication examined, we cannot be confident about the proportion of use of these drugs outside a research context.

In this systematic review, we were especially interested in evidence for non-pharmacological treatment in musculoskeletal sarcoidosis. Physical training or pulmonary rehabilitation (PR) is an important element of the comprehensive care of people with chronic diseases, including musculoskeletal disorders. The lack of studies with systematic assessment of rehabilitative measures in musculoskeletal sarcoidosis is another important knowledge gap.

To collect information on the benefits of physical activity and training in sarcoidosis, Strookappe et al. [55] performed a comprehensive literature review. As might be expected, all selected publications involved the pulmonary organ and only one was an RCT [56]. One reason for this lack of published evidence may be that most patients with Löfgren’s syndrome have a good prognosis and are initially not in need of physical therapy. Nonetheless, the absence of effect studies on non-pharmacological therapy of musculoskeletal sarcoidosis is striking.

This is, to our best knowledge, the first systematic review on pharmacological and non-pharmacological treatment of musculoskeletal sarcoidosis. A further strength is the application of a systematic literature search and quality assessment of the included studies. A major limitation of this review is that the studies had a high heterogeneity in included patients, and did not always differentiate clearly between musculoskeletal and pulmonary outcomes. Second, the methodological quality of the included studies was poor. Finally, our review was not registered in a prospective register of systematic reviews.

In conclusion, findings in our review do not allow guidance on the effect of pharmacological and non-pharmacological treatment of musculoskeletal manifestations in sarcoidosis. Future studies should use randomised controlled designs and include homogeneous patient samples.

## Appendix 1

See Table 2.

**Table 2:** Search strategy for medline and embase

Database/source	Ovid MEDLINE
Search date	March 29, 2022
Search history	Database: Ovid MEDLINE(R) and Epub Ahead of Print, In-Process, In-Data-Review & Other Non-Indexed Citations and Daily < 1946 to March 28, 2022 >
	Search Strategy:
	––––––––––––––––––––––––––––––––––––––––
	1 Sarcoidosis/
	2 Musculo-skeletal System/
	3 exp muscles/
	4 exp "Bone and Bones"/
	5 exp Joints/
	6 exp Skeleton/
	7 or/2–6
	8 and/1,7
	9 (sarcoid* adj2 bone*).mp
	10 (sarcoid* adj2 joint*).mp
	11 (sarcoid* adj2 musc*).mp
	12 (sarcoid* adj2 skelet*).mp
	13 (sarcoid* adj2 arthri*).mp
	14 (rheuma* adj2 sarcoid*).mp
	15 (lofgren* adj2 syndrome).mp
	16 or/9–15
	17 or/8,16
	18 exp Rehabilitation/
	19 rehabilit*.mp
	20 exp Exercise Therapy/
	21 exercise*.mp
	22 non-pharmac*.mp
	23 exp Therapeutics/
	24 therapeutic*.mp
	25 exp Prednisolone/
	26 prednisolone.mp
	27 exp corticosteroid/
	28 corticosteroid*.mp
	29 exp Adrenal Cortex Hormones/
	30 adrenal cortex.mp
	31 corticoid*.mp
	32 exp Glucocorticoids/
	33 glucocorticoid*.mp
	34 exp Budesonide/
	35 budesonide.mp
	36 exp Methotrexate/
	37 methotrexate.mp
	38 exp Leflunomide/
	39 leflunomide.mp
	40 exp Azathioprine/
	41 azathioprine.mp
	42 exp Tumor Necrosis Factor-alpha/
	43 "Tumor Necrosis Factor".mp
	44 TNF*.mp
	45 exp Infliximab/
	46 infliximab.mp
	47 exp Adalimumab/
	48 adalimumab.mp
	49 exp Mycophenolic Acid/
	50 mycophenolic acid.mp
	51 or/18–50
	52 exp "Systematic Review"/
	53 (systematic* adj2 review).mp
	54 exp Randomized Controlled Trial/
	55 ((randomized or randomised) adj2 (controlled or trial)).mp
	56 ((cluster or quasi) adj2 (randomized or randomised)).mp
	57 stepped wedge.mp
	58 exp Regression Analysis/
	59 (regression adj2 (discontinuity or analys* or design or study)).mp
	60 (controlled adj2 (clinical or study or trial)).mp
	61 exp Longitudinal Studies/
	62 (longitudinal adj2 (study or trial or design)).mp
	63 exp Cohort Studies/
	64 (cohort adj2 (study or trial or analysis)).mp
	65 case series.mp
	66 or/52–65
	67 Treatment Outcome/
	68 ((effect* or efficac* or outcome* or response) adj4 (treatment* or therap* or intervention or rehabilitation)).mp
	69 or/67–68
	70 or/66,69
	71 and/17,51,70
	***************************
Hits	219
Duplicates (removed)	1

Database/source	Embase
Search date	March 29, 2022
Search history	Database: Embase < 1974 to 2022 March 28 >
	Search Strategy:
	––––––––––––––––––––––––––––––––––––––––
	1 Sarcoidosis/
	2 musculoskeletal system/
	3 exp muscle/
	4 exp joint/
	5 exp skeleton/
	6 exp bone/
	7 or/2–6
	8 and/1,7
	9 (sarcoid* adj2 bone*).mp
	10 (sarcoid* adj2 joint*).mp
	11 (sarcoid* adj2 musc*).mp
	12 (sarcoid* adj2 skelet*).mp
	13 (sarcoid* adj2 arthri*).mp
	14 (lofgren* adj2 syndrome).mp
	15 or/9–14
	16 or/8,15
	17 exp Rehabilitation/
	18 rehabilit*.mp
	19 exp kinesiotherapy/
	20 exercise*.mp
	21 non-pharmac*.mp
	22 exp therapy/
	23 exp Prednisolone/
	24 prednisolone.mp
	25 exp corticosteroid/
	26 corticosteroid*.mp
	27 adrenal cortex.mp
	28 exp adrenal cortex/
	29 corticoid*.mp
	30 exp Glucocorticoids/
	31 glucocorticoid.mp
	32 exp Budesonide/
	33 budesonide.mp
	34 exp Methotrexate/
	35 methotrexate.mp
	36 exp Leflunomide/
	37 leflunomide.mp
	38 exp Azathioprine/
	39 azathioprine.mp
	40 exp Tumor Necrosis Factor/
	41 "Tumor Necrosis Factor".mp
	42 TNF*.mp
	43 exp Infliximab/
	44 infliximab.mp
	45 exp Adalimumab/
	46 adalimumab.mp
	47 exp Mycophenolic Acid/
	48 mycophenolic acid.mp
	49 or/17–48
	50 exp "systematic review"/
	51 (systematic adj2 review).mp
	52 exp Randomized Controlled Trial/
	53 ((randomized or randomised) adj2 (controlled or trial)).mp
	54 ((cluster or quasi) adj2 (randomized or randomised)).mp
	55 stepped wedge.mp
	56 exp Regression Analysis/
	57 (regression adj2 (discontinuity or analys* or design or study)).mp
	58 (controlled adj2 (clinical or study or trial)).mp
	59 exp longitudinal study/
	60 (longitudinal adj2 (study or trial or design)).mp
	61 exp Cohort Analysis/
	62 (cohort adj2 (study or trial or analysis)).mp
	63 case series.mp
	64 or/50–63
	65 Treatment Outcome/
	66 ((effect* or efficac* or outcome* or response) adj4 (treatment* or therap* or intervention or rehabilitation)).mp
	67 or/65–66
	68 or/64,67
	69 and/16,49,68
	***************************
Hits	574
Duplicates (removed)	68

## Appendix 2

See Table 3.

**Table 3:** Excluded articles (*n* = 30)

Author/year	Design	Population	Aims/intervention	Outcomes	Reason for exclusion
Androdias 2011 [27]	Retrospective case-series	CNS sarcoidosis/sarcoid myopathy (*n* = 10) Neurosarcoidosis	Mycophenolate mofetil	Remission, relapse, clinical and radiological improvement, side effects	Wrong population
Banse 2013 [28]	Retrospective study	Joint manifestations of sarcoidosis	TNF inhibitors	Efficacy & safety	Abstract
Barnard 2001 [29]	Non-systematic review	Sarcoidosis	First line: corticosteroids. Alternatives: methotrexate, cyclosporin A, Azathioprine, chloroquine, hydroxychloroquine + +	Not specified	Review
Bello 2014 [30]	Systematic review + case report	12 different joint diseases	Intra-articular TNF inhibitors	Several	Review Case report
Ben-Hassine 2019 [31]	Retrospective multi-center study	Sarcoidosis	Glucocorticoids, methotrexate, hydroxychloroquine	Several	Case–control
Crommelin 2015 [32]	Letter to editor	Joint involvement in sarcoidosis	Prednisone, NSAIDs, methotrexate	Not specified	Letter to editor
Glanowski 2018 [33]	Retrospective multi-center study	Sarcoidosis with bone involvement	Prednisone n = 8 HCQ *n*= 8 MTX *n* = 5	Complete remission Partial remission	Abstract
Hammam 2020 [9]	Registry study	Sarcoidosis	Several	Pharmacological treatments	No specific results for muskulo-skeletal sarcoidosis
Hassine 2018 [34]	Case–control	Sarcoidosis with bone manifestations	Glucocorticoids (*n* = 63) MTC (*n* = 12) HCQ (*n* = 6	Thoracic and extra-thoracic lymph node, pulmonary, cutaneous involvement and hypercalcemia	Abstract
Hobbs 2005 [35]	N = 1	Chronic sarcoid arthritis	Intra-articular etanercept	Pain, stiffness, physical disabilities associated with synovitis	Abstract Case study Letter to editor
Huang 2011 [36]	N = 1	Skeletal and lymphoreticular sarcoidosis Polyarticular sarcoidosis	High-dose corticosteroids and methotrexate + infliximab	Early morning stiffness, swollen and tender joint count, Health Assessment Questionnaire	Abstract
James 1976 [37]	Non-systematic review	Sarcoidosis with bone and joint involvement	Mainly about prevalence, prognosis and diagnostics	Several	Review
Johns 1986 [38]	Longitudinal study	Chronic sarcoidosis	Outcomes and complications after corticosteroid therapy	General prognosis	Not separate results for musculoskeletal manifestations
Khanna 2003 [39]	*N* = 1	Chronic sarcoidosis arthropathy and lupus pernio	Etanercept	Morning stiffness, skin lesions, tenderness, radiograph	Case report
Madronal-Garcia [40]	Retrospective descriptive study	Sarcoidosis with joint manifestations	Corticosteroids/ immunosuppressants	Prognosis/ need of treatment	Abstract
Mana 2017 [41]	Case series	Sarcoidosis	Corticosteroids, steroid-sparing agents	Prognosis	No specific results for muskulo-skeletal sarcoidosis
Martin Varillas 2019 [42]	Retrospective cohort	Sarcoidosis (systemic) sarcoidosis	TNF inhibitors	Several	Abstract
Mathur 1993 [43]	Non-systematic review	Acute and chronic sarcoidosis	NSAIDs, prednisone, ketoconazole, deflazacort, chloroquine	Hypercalcemia, hypercalciuria	Review
Medici 1977 [44]	Retrospective cohort study	Löfgren’s syndrome	To determine whether steroid therapy affects the course and prognosis	Duration of acute illness, side effects	Abstract
Neville 1983 [45]	Retrospective cohort study	Histologically proven sarcoidosis	To provide a prognostic index for each clinical manifestation of sarcoidosis	Chest resolution and sarcoidosis remission in 2 years	No information of treatments received
Oshagbemi 2017 [46]	National Registry study	Sarcoidosis	Oral glucocorticoids	Osteoporotic fractures	Case–control
Patil 2018 [47]	Retrospective study	Musculo-skeletal Sarcoidosis (14.8% Lofgren + chronic arthritis + osseous myopathy)	Prednisone methotrexate, TNF inhibitors	Prevalence of sarcoidosis/use of drugs	Abstract
Retamozo 2019 [48]	National registry study	Musculo-skeletal sarcoidosis	Glucocorticoids/ immunosuppressive agents	Clinical presentations	Abstract
Rubio-Rivas 2019 [49]	Retrospective cohort study	Sarcoidosis	To compare young and old patients	Clinical features and prognosis	No specific results for muskulo-skeletal sarcoidosis
Rubio-Rivas 2020 [5]	Retrospective cohort study	Sarcoidosis	To compare patients with and without Löfgren’s syndrome	Epidemiological, clinical, radiological and prognostic features	No specific results for muskulo-skeletal sarcoidosis
Russell 2013 [50]	One group before-after study	Multi-organ sarcoidosis (pulmonary and non-pulmonary)	Infliximab	Disease activity, adverse events	No specific results for muskulo-skeletal sarcoidosis
Schmajuk 2019 [51]	National registry study	Sarcoidosis	Several	Medication use	Abstract
Somayeh 2019 [52]	Non-systematic narrative review	Musculo-skeletal manifestations	(1)NSAIDS, Corticoids (2)Methotrexate, stereohydroxychloroquine, azathioprine, leflunomide (3)TNF inhibitors	Not specified	Review
Tejera Segura 2014 [53]	Retrospective case series	Löfgren’s syndrome	(4)Describe clinical characteristics, treatment and outcome	Subdiagnostics, biopsies, treatments	Language
Zhou 2017 [54]	Medical record review	Bone sarcoidosis	Infliximab	Clinical characteristics	Case–control

## Appendix 3

See Table 4.

**Table 4:** Appraisal of methodological quality

Study/year	1. Clear inclusion criteria?	2. Condition measured in standard, reliable way?	3. Valid methods used for identification of the condition?	4. Consecutive inclusion?	5.Complete Inclusion?	6. Clear reporting of demographics?	7. Reporting of clinical information	8. Outcomes or follow-up Clearly reported?	9. Clear reporting of the presenting site(s)/clinic(s) demographic information?	10. Statistical analysis appropriate?
Arthritis in Sarcoidosis Group 2018	Yes	Yes	Yes	Unclear	Unclear	Yes	Yes	Yes	Yes	Yes
Banse 2013	Yes	Yes	Yes	Yes	Yes	Yes	Yes	Yes	No	Yes
Cacciatore 2020	Yes	Yes	Yes	Unclear	Unclear	Yes	Yes	Yes	No	yes
Fayad 2006	Yes	Yes	Unclear	Yes	Yes	Yes	Yes	Yes	No	Yes
Garg 2009	Yes	Yes	Yes	Yes	Unclear	No	Unclear	No	No	No
Glennås 1995	Yes	Yes	Yes	Yes	Yes	Yes	Yes	Yes	Yes	Yes
Loupasakis 2015	Yes	Yes	Unclear	Yes	Yes	Yes	Yes	Unclear	Yes	Yes
Mana 2003	Yes	Yes	Yes	Yes	Yes	Yes	Yes	Yes	Yes	Yes
Miller 2019	No	Unclear	Unclear	No	Unclear	Yes	Yes	Yes	No	Yes
Perruquet 1984	Yes	Unclear	Unclear	No	Yes	Yes	Yes	No	Yes	Unclear
Petursdottir 2007	Yes	Unclear	Unclear	No	No	Yes	Yes	No	No	Unclear
